# Examining the Impact of Brief Mindfulness Practice on Sustained Attention, Attentional Inhibition and Convergent Thinking

**DOI:** 10.3390/jintelligence13090119

**Published:** 2025-09-16

**Authors:** Zoe D. Hughes, Linden J. Ball, Petar Atanasov, Jeannie Judge

**Affiliations:** 1 School of Engineering and Computing, University of Lancashire, Fylde Road, Preston PR1 2HE, UK; zdhughes1@lancashire.ac.uk; 2 Liverpool John Moores University, Liverpool L3 2AJ, UK; 3 School of Psychology & Humanities, University of Lancashire, Fylde Road, Preston PR1 2HE, UK; pfatanasov@lancashire.ac.uk (P.A.); jeanne.judge107@gmail.com (J.J.)

**Keywords:** mindfulness, sustained attention, attentional inhibition, creative cognition, rebus puzzles

## Abstract

There remains little understanding of how short-term mindfulness interventions influence creative cognition. We report an experiment that examined the impact of a brief mindfulness intervention on sustained attention, attentional inhibition, and convergent thinking, relative to a control group. Participants (*N* = 117) were assigned to either a brief mindfulness practice (*n* = 60) or an active control task (*n* = 57), before completing the following: (i) a Sustained Attention to Response Task (SART), to assess sustained attention; (ii) a flanker task, to assess attentional inhibition; and (iii) a convergent thinking task (a series of rebus puzzles). The mindfulness group showed faster reaction times than the control group on the SART, along with fewer task-unrelated mind-wandering thoughts, suggestive of better sustained attention. The mindfulness group also demonstrated improved reaction times and accuracy relative to the control group during the flanker task, indicating enhanced inhibitory control. However, rebus puzzle scores did not differ between groups, indicating that although a brief mindfulness practice enhances sustained attention and attentional inhibition, this improved attentional control does not facilitate convergent thinking in solving rebus puzzles.

## 1. Introduction

Mindfulness practice, defined as paying attention to the present moment without feelings of judgement or overwhelm (Kabat-Zinn 1982), has received growing interest because of the potentially advantageous outcomes that mindfulness-based interventions (MBIs) afford for everyday tasks. More specifically, many researchers have argued that mindfulness practice exerts beneficial effects on different aspects of attention, including sustained attention, inhibitory control, and cognitive flexibility (for a recent review, see Verhaeghen 2021). These claimed benefits are perhaps unsurprising given that attentional mechanisms are proposed to be core to engaging in mindfulness practice and necessary to improve non-judgemental awareness, overall self-regulation, and positive behavioural outcomes (Lindsay and Creswell 2017; Tang et al. 2015). Although the terminology involved in models of mindfulness has differed over the past decade (e.g., encompassing notions such as awareness, cognitive flexibility, emotional regulation and acceptance), it is nevertheless the case that the concept of attention continues to be pivotal for understanding the mechanisms that are engaged by mindfulness practice. As such, it seems important to examine further the role that attention plays in influencing how mindfulness impacts cognition, and to extend these investigations to advancing an understanding of the benefit of mindfulness for important real-world activities such as creative cognition. The research reported in the present paper aimed to address these issues, with a specific focus on the potentially positive impact of brief mindfulness practice on sustained attention, attentional inhibition and creative performance, as well as the mediating role played by attentional mechanisms for creativity outcomes.

It is noteworthy that in the literature, attentional mechanisms such as executive functioning are proposed to be highly associated with creative thinking processes (Sharma and Babu 2017). Creative thinking is often conceptualised as comprising two core components: (i) divergent thinking, the ability to generate multiple novel ideas or solutions; and (ii) convergent thinking, the ability to identify a single, appropriate solution to a problem (Guilford 1967; Runco and Acar 2012). These components are not equivalent to creativity itself but are widely accepted as being key indices or cognitive markers of the creative process. Divergent thinking is often assessed through tasks that measure the fluency, flexibility, and originality of idea generation (e.g., the Alternative Uses Task, where participants are asked to come up with novel uses for a common, everyday object), while convergent thinking is typically assessed through tasks requiring insight and precision, such as the Compound Remote Associates Test or rebus puzzles, which have also been argued to tap into insight-based problem solving (Lee and Therriault 2013; Salvi et al. 2016).

Recent research has started to disentangle how mindfulness might influence these distinct components of creative thinking. For example, mindfulness has been associated with enhanced divergent thinking via mechanisms such as increased openness to experience and emotional regulation. Giancola et al. (2024) demonstrated that both cognitive reappraisal and dispositional mindfulness mediated the positive relationship between openness and divergent thinking, highlighting the affective and attentional pathways through which mindfulness may support idea generation. However, other studies have pointed to possible benefits of mindfulness for convergent thinking, particularly through improved executive attention and cognitive control (e.g., Ostafin and Kassman 2012; Zedelius and Schooler 2016).

The seemingly central role of attention in both mindfulness practice and creative cognition raises an important question as to whether mindfulness can facilitate convergent or divergent thinking through attentional enhancement. Although some meta-analytical reviews (e.g., Lebuda et al. 2016) support a general positive association between mindfulness and creative thinking, the field still lacks consensus on the generalisability and underlying mechanisms of these effects. Indeed, a more recent meta-analysis by Hughes et al. (2023), which presents an extensive and systematic review of the relationship between mindfulness and creative thinking, demonstrates stronger and more consistent positive effects of mindfulness on convergent thinking than on divergent thinking. This supports the notion that attentional mechanisms enhanced by mindfulness may be more directly involved in convergent rather than divergent thinking tasks, with the former typically requiring greater cognitive control and goal-directed attention (Hughes et al. 2023).

Given this emerging pattern of evidence, the present study focuses specifically on convergent thinking as a key outcome of mindfulness practice. In doing so, we adopt the position that convergent thinking is one measurable index of creative cognition, and that it may benefit from the attentional enhancements provided by mindfulness. The current experiment therefore investigates whether the attentional pathways associated with mindfulness (e.g., sustained attention and attentional inhibition) can contribute to enhanced convergent thinking performance, and whether these attentional mechanisms mediate this effect.

### 1.1. Mindfulness and Attention

Mindfulness has been associated with improved performance on tasks that measure attention, regardless of the length of practice (e.g., Mak et al. 2018; Verhaeghen 2021). However, the picture is complicated because of the range of attention-related tasks that have been utilised across studies. One attention-related construct that has been subjected to particularly close scrutiny in the mindfulness literature is that of sustained attention (e.g., Verhaeghen 2021; Wimmer et al. 2020), which is the ability to focus on an activity or stimulus over a long period of time (Oken et al. 2006). Extensive findings are consistent with the view that mindfulness can serve as an effective tool to improve people’s ability to sustain attention (Schuman-Olivier et al. 2020), as evidenced by people’s higher accuracy and faster reaction times following mindfulness practice on classic tasks such as the Sustained Attention to Response Task (SART), where participants must respond to frequent non-target stimuli while withholding responses to rare target stimuli, thus also engaging in a degree of attentional inhibition (Bauer et al. 2020).

Several studies have also focused very directly on examining the influence of mindfulness practice on attentional inhibition, a key executive function involving the ability to suppress irrelevant or interfering stimuli. As an umbrella term, inhibition is the suppression of covert responses to prevent incorrect overt responses from arising (Verbruggen and Logan 2009) and can be measured using tasks like the flanker task (Eriksen and Eriksen 1974; Robertson et al. 1997), which is described later, and the Stroop colour-word task (cited in Stroop 1935), which involves naming the colour in which a word is written while ignoring the word itself, which typically represents the name of a conflicting colour. For example, Jensen et al. (2012) used the Stroop colour-word task to measure attention inhibition in the following groups: (i) a mindfulness group; (ii) an active control group, where participants engaged in the Health Enhancement Programme, a non-meditative intervention incorporating activities such as physical exercise, music therapy, and nutritional education designed to match the mindfulness programme in structure and time commitment without involving meditation; and (iii) an inactive control group, where participants received no comparison treatment during the study (also referred to as a no-treatment control group; see Kinser and Robins 2013). Jensen et al. (2012) reported significant improvements in performance on the Stroop task in the mindfulness group compared to both control groups following a long-term, eight-week mindfulness course.

Positive outcomes on attentional inhibition have also been reported from mindfulness practice utilising conflict paradigms like the flanker task, developed by Eriksen and Eriksen (1974), which requires participants to focus on a central target while ignoring potentially distracting flankers. Critical to the flanker task is the occurrence of “incongruent” trials, where flankers are mapped to the opposite response category to the central target, thereby creating conflict (e.g., < < > < <), as opposed to “congruent “trials, where the flankers are mapped to the same response category as the central target (e.g., < < < < <). This manipulation typically results in slower reaction times and lower accuracy rates for incongruent relative to congruent trials (Heuer et al. 2023; Kałamała et al. 2018), with potential explanations rooted in conflict monitoring and cognitive control theories (Botvinick et al. 2001; Carter and Van Veen 2007), along with strategic influences, including the “utility principle”, which suggests that individuals allocate cognitive resources based on the anticipated costs and benefits of their responses (e.g., Gratton et al. 1992; Lehle and Hübner 2008). Studies using the flanker task have shown that mindfulness interventions can improve performance on both congruent and incongruent trials, which has been taken to indicate enhanced inhibition and executive attention (Jha et al. 2007; Norris et al. 2018). However, not all studies replicate these findings, with the suggestion being that outcomes may be influenced by moderating factors, such as baseline mood, levels of stress, overall wellbeing, and personality characteristics, including conscientiousness or openness (Larson et al. 2013; Lin et al. 2019).

The flanker task has been used extensively by cognitive researchers for many years and has been modified in various ways to manipulate task demands, including through the use of dual-task conditions (e.g., Hogg et al. 2022) and alterations in stimulus timing (e.g., Melara et al. 2018). One intriguing manipulation is the use of “reversed” trials in which participants must respond with the “opposite” key to that required for the standard response for congruent and incongruent trials. Participants are alerted to reversed trials when the arrow stimuli are presented in a different colour to the standard trials (see Section 2.3.2 for additional detail and an example figure). Reversed trials introduce an additional level of difficulty (Bugg 2008; Simon and Wolf 1963), because they require interference suppression and response inhibition, such that a previous stimulus-response mapping needs to be suppressed. Although few studies have employed the flanker paradigm with this type of modification, there is evidence that reversed flanker trials increase task demands, thereby leading to slower reaction times compared to standard congruent and incongruent trials (Richardson et al. 2018). The use of reversed trials adds an additional layer of cognitive load by requiring participants to override automatic response tendencies, which arguably mimics real-world scenarios more accurately (Braver et al. 2009). Furthermore, incorporating reversed trials can help to differentiate between basic reaction time improvements and genuine enhancements in cognitive flexibility and control mechanisms (Egner 2008; Hommel 2011). As such, in the study that we report below we decided to include reverse trials alongside congruent and incongruent trials.

To conclude this section, we note that although there seems to be ample evidence to support the effectiveness of mindfulness practice in improving attentional processes on the SART as well as the flanker task, it remains important to acknowledge that these tasks differ fundamentally in their cognitive demands. Modifications such as reversed trials add to the complexity that more closely mimics real-world scenarios that require interference suppression and response inhibition. By incorporating such tasks and modifications, the present research seeks to build on prior findings and provide a more nuanced understanding of how mindfulness practice influences performance across distinct attentional domains.

### 1.2. Attention and Convergent Thinking

There is extensive evidence suggesting that creative cognition is reliant on executive and sustained attention (De Dreu et al. 2012; Gilhooly et al. 2015). Specifically, convergent thinking—the process of identifying a single creative solution—has been found to correlate with working memory capacity (WMC; Lee and Therriault 2013; Takeuchi et al. 2020), which reflects an individual’s ability to maintain focus on a task while suppressing distracting or irrelevant thoughts (Keller et al. 2019). Convergent thinking therefore relies heavily on the “executive control network”, which facilitates the focus required to narrow down possibilities to a single, optimal solution (Beaty et al. 2015).

**Figure 1:**
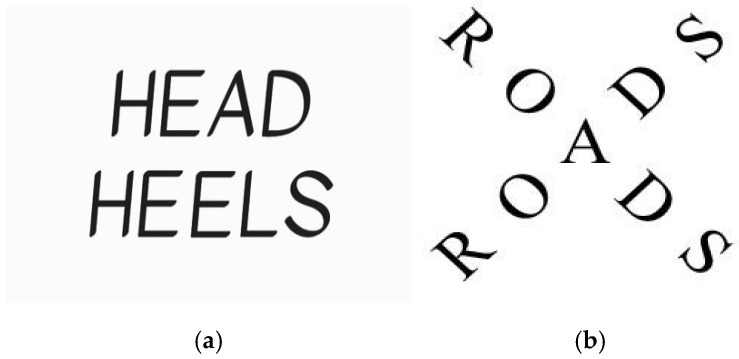
Examples of rebus puzzles. *Note:* (**a**) the word “HEAD” is placed above “HEELS”, representing the phrase “Head over heels”. (**b**) depicts two roads crossing each other, forming an intersection, representing the phrase “Crossroads”.

A common method for measuring convergent thinking is to present participants with a series of rebus puzzles to solve, whereby each puzzle requires a phrase or saying to be deciphered from a combination of visual, spatial, verbal, or numerical cues (Threadgold et al. 2018). As illustrated in Figure 1, each rebus puzzle has a single correct solution. Sustained attention is essential when attempting such puzzles to resist attentional drift while synthesising the various presented cues into a coherent representation and a possible solution response (Robertson et al. 1997). In other words, sustained attention minimises errors from lapses in concentration, thereby acting as a stabilising force during the problem-solving process (Unsworth et al. 2014). Furthermore, attentional inhibition is also central to the solution process with rebus puzzles to enable the filtering out of distractions and competing stimuli as well as task-irrelevant representations (Beaty and Silvia 2012). In this way, inhibitory control ensures the maintenance of a task-relevant cognitive environment, which is crucial for preventing cognitive overload and for refining a broad array of possibilities into a single, actionable solution (Benedek et al. 2014).

In sum, it would appear that sustained attention and attentional inhibition work together to enable people to solve convergent creative problems such as rebus puzzles. The present study focuses on the outcomes of mindfulness for these two core attentional processes and seeks to understand how these processes may be linked to convergent thinking, as assessed using rebus puzzles.

### 1.3. Overview of the Current Experiment

As we have discussed, extensive research has examined the cognitive advantages of mindfulness as a long-term structured experience consisting of repeated daily practice (e.g., in the form of a multi-week, mindfulness-based stress reduction course; Hargus et al. 2010). Such studies have provided a good level of support for the benefits of long-term mindfulness interventions on sustained attention (Bauer et al. 2020) and attentional inhibition (Prakash et al. 2020). More recently, however, researchers have shown increased interest in examining the potentially beneficial outcomes of short-term mindfulness practice (i.e., a single session) on attentional processes, thereby avoiding the need for time-consuming, extensive and expensive long-term interventions. This has led to shorter-term mindfulness interventions, sometimes referred to as “inductions”, receiving more focus in the recent literature.

We define short-term mindfulness interventions as being those that take place over one session only. The timeframe of the sessions that have been used in the literature are seen to vary from as little as 10 min (Norris et al. 2018) up to 90 min (Wimmer et al. 2020), but the critical defining features of short-term interventions is that they involve neither breaks nor follow-up training sessions. When we consider these short interventions, there has been very little interest in their use until recently, perhaps due to reservations that such brief interventions are unlikely to produce any worthwhile outcomes on cognition (Prakash et al. 2020). Surprisingly, though, research has started to reveal that interventions of only 10 min can elicit the same kinds of beneficial outcomes on cognition, including attentional control tasks, that are observed from long-term courses (Norris et al. 2018; Thompson et al. 2021). However, despite some limited evidence, the literature investigating interventions of less than 20 min remains sparse. The current experiment therefore builds upon the well-documented associations between mindfulness practice and its benefits for sustained attention and attentional inhibition (e.g., Chiesa et al. 2011; Whitfield et al. 2022), but with a specific focus on short mindfulness practice and the role of any resulting benefits of enhanced attentional processes for creative cognition, as measured in terms of convergent thinking performance.

To assess sustained attention, we employed the Sustained Attention to Response Task (SART) with embedded thought probes. This task not only measures participants’ ability to maintain focus over time but also provides insight into the prevalence of “mind-wandering” through the use of the thought probes that serve as a proxy for attentional lapses, which mindfulness practice is reported to reduce (Verhaeghen 2021). The SART’s relatively low cognitive demand makes it ideal for examining mind-wandering episodes. Research has consistently shown that lower task demands correlate with higher frequencies of mind wandering, as attentional resources are less fully engaged (e.g., Brosowsky et al. 2021). By incorporating thought probes, we aimed to directly measure participants’ self-reported attention during the task, providing a nuanced understanding of how brief mindfulness interventions influence sustained attention and whether this is evidenced by mitigated task-irrelevant thoughts.

To assess attentional inhibition in our experiment, we adopted a flanker task. As we discussed above, this task requires participants to focus on a central target while ignoring distracting flankers. The inclusion of both congruent and incongruent trials, alongside novel “reversed” trials, enabled a detailed examination of inhibitory control under varying levels of cognitive demand. The outcomes of this task are particularly relevant for understanding how mindfulness practice may enhance an individual’s ability to suppress irrelevant stimuli and resolve cognitive conflict, both of which are essential for successful convergent thinking.

We predicted that on the SART, the mindfulness group would be more accurate, reflecting enhanced sustained attention and less task-irrelevant thoughts caused by mind wandering (as measured using mind-wandering probes). Turning to the flanker task, we predicted that participants in the mindfulness group would exhibit faster reaction times and higher accuracy compared to the control group, particularly for incongruent trials, which require attentional inhibition, as well as for reversed trials, which require the inhibition of a previously associated response mapping. In congruent trials, although both groups would be expected to perform well, we reasoned that the mindfulness group may demonstrate improved performance due to improved attentional focus. In relation to our convergent thinking measure, we expected that the mindfulness group would have higher accuracy on rebus puzzles, drawing on evidence that mindfulness practice improves convergent thinking through enhanced sustained attention and attentional inhibition, which are essential for the process of generating single correct responses in convergent thinking tasks. We planned to assess the role of sustained attention and attentional inhibition in mediating the link between mindfulness and creative performance by undertaking a statistical mediation analysis. In summary, we hypothesise the following:

(i) Sustained attention and attentional inhibition will be higher in the mindfulness group relative to the control group.
(ii) Convergent thinking performance will be higher in the mindfulness group relative to the control group.
(iii) Attentional improvements will mediate any observed link between mindfulness and convergent thinking.

## 2. Methods

### 2.1. Participants

A power calculation (Faul et al. 2007) determined that a sample of 108 participants was required with power set at 0.8, alpha set at 0.05, and an expected medium effect size (*d* = 0.6). Thus, a sample size of 125 participants was selected based on expectations of a medium effect size, commensurate with prior research (e.g., Rahl et al. 2017). Eight participants were excluded due to technical errors and performance under 10% accuracy. A total of 117 participants (*n* = 60 in the mindfulness condition) were included in the final analyses, comprising undergraduate and postgraduate students from the University of xxxxx. Among them, 89 participants were female and 104 were right-handed, with an age range of 18 to 51 years (*M* = 22.56, *SD* = 6.2). All participants reported normal or corrected-to-normal visual acuity and hearing, while also being fluent in English and proficient in reading and writing. The participant sample was predominantly White British (*n* = 81; 69.2%), and 21 participants reported previous mindfulness experience.

Group-level demographic information is reported in Appendix A. The mindfulness group (*n* = 60) included 46 females and 12 males (2 undisclosed), with a mean age of 23.02 years (*SD* = 7.07); 38 identified as White British. The control group (*n* = 57) included 43 females and 14 males, with a mean age of 22.09 years (*SD* = 5.20); 43 identified as White British. Between-group comparisons revealed no significant differences in age, gender, handedness, or previous mindfulness experience (all *p*s > 0.1; see Appendix B), suggesting demographic equivalence between groups. Participants gave written informed consent and were awarded course credits, where relevant, as well as a £10 Amazon voucher incentive as compensation for their time.

### 2.2. Design

The experiment employed an independent measures design to determine whether a brief mindfulness intervention influenced sustained attention, attentional inhibition and convergent thinking. The independent variable was group (mindfulness intervention vs. active control) and the key dependent variables were accuracy and reaction times in the SART and flanker task, and accuracy in solving the rebus puzzles. When examining accuracy and reaction times in the flanker task, we treated congruent, incongruent and reversed trials as being three levels of an independent variable that we refer to as stimulus type. Participants were pseudo-randomly assigned to either a 10 min mindfulness intervention, or a 10 min excerpt from an audio book as a validated active control task (cf. MacCoon et al. 2012). Group assignment was based on the order in which participants registered for the study: those with odd participant numbers were allocated to the mindfulness group, and those with even participant numbers were allocated to the control group.

### 2.3. Materials

Both the SART and flanker task were presented using *E*-Prime (Version 2.0.10; Psychology Software Tools, Pittsburgh, PA, USA) and displayed on a 14-inch LCD screen in 30-point Times New Roman font as black text on a white background. At 55 cm viewing distance, each character subtended approximately 1° of visual angle and represented a normal size for reading. The figural rebus puzzle task was administered via Qualtrics (Provo, UT, USA) with each puzzle presented centrally and in full-screen mode.

#### 2.3.1. The SART

Sustained attention was assessed using the SART (Robertson et al. 1997), a computer-based go/no-go task. Participants were instructed to press the space bar in response to frequent non-target digits (“1”, “2”, “4”, “5”, “6”, “7”, “8”, and “9”), which appeared in 89% of trials, and to withhold their response to a single infrequent target digit (“3”), which appeared in 11% of trials. Each digit was displayed centrally on the screen for 200 ms against an off-white background, followed by a 900 ms mask before the next trial. The task comprised 225 trials in total (25 of each digit) presented in a random sequence and took approximately 8 min to complete, consistent with previous research protocols (e.g., Manly et al. 1999).

**Figure 2:**
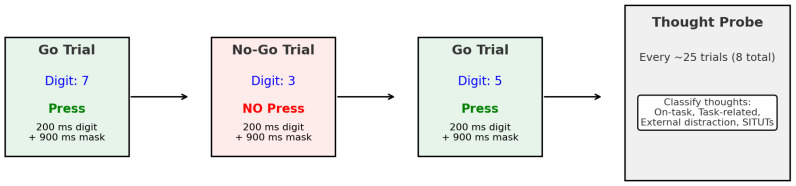
The task design to measure thought probes during the Sustained Attention to Response Task (SART).

To capture fluctuations in sustained attention, thought probes were intermittently embedded within the SART. These probes were randomly presented once every 25 trials, resulting in eight probes across the task, aligning with established methodologies (e.g., Jackson and Balota 2012). When presented with a probe, participants classified their thoughts based on Stawarczyk et al.’s (2011) multidimensional framework: (i) on-task thoughts; (ii) task-related thoughts; (iii) external distraction thoughts; (iv) stimulus-independent and task-unrelated thoughts (SITUTs)—see Figure 2 for a depiction of the task design.

#### 2.3.2. The Flanker Task

Attentional inhibition was assessed using a modified flanker task (Eriksen and Eriksen 1974), which involved 438 trials. These trials were equally divided into three types: 146 congruent, 146 incongruent, and 146 reversed, with each type containing an equal number of leftward and rightward target arrows (73 each). In congruent trials, participants viewed a horizontal array of five green arrows in which the central target arrow was flanked on either side by arrows pointing in the same direction. In incongruent trials, the flankers again appeared in green but pointed in the opposite direction to the central arrow, creating a conflict that required greater attentional control. Reversed trials were visually distinguished by red arrow stimuli and followed a different rule: participants were instructed to respond in the opposite direction to that indicated by the central target arrow. We did not distinguish between congruency in reversed trials because, under the reversed response mapping, trials labelled as “incongruent” would still feature four out of five arrows pointing in the same direction, making them perceptually like congruent trials and thus unlikely to differ meaningfully in processing. To verify our assumption, we compared reversed incongruent and reversed congruent trials and found no significant differences in accuracy or reaction times in the flanker task, supporting our decision to collapse across congruency in reversed trials. Detailed analyses are provided in Appendix C.

**Figure 3:**
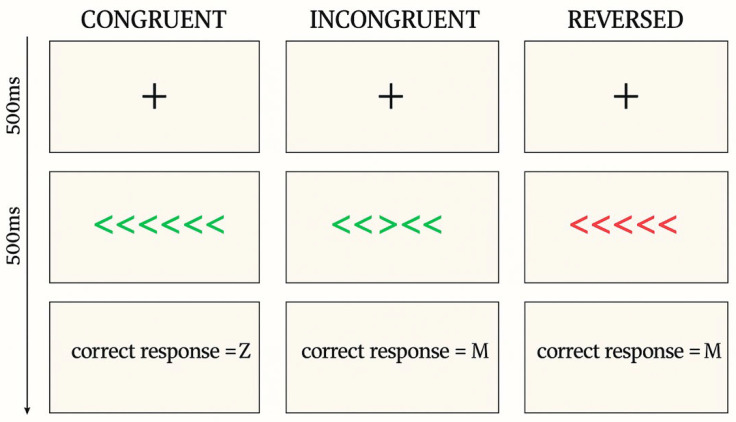
Graphical representations of the congruent, incongruent and reversed flanker trials with correct responses.

Responses were recorded using the computer keyboard, with participants pressing the “Z” key for a leftward response and the “M” key for a rightward response. On congruent and incongruent (green) trials, responses corresponded to the direction of the central arrow. On reversed (red) trials, participants were required to respond in the opposite direction (i.e., pressing “M” for a leftward-pointing arrow and “Z” for a rightward-pointing arrow). Each trial began with a central fixation cross presented for 500 ms, followed by the stimulus display for 500 ms, and then a blank screen for another 500 ms. Figure 3 shows the trial set-up with correct responses. Trials were terminated either by a participant response or after 500 ms had elapsed without a response. To ensure comprehension, participants completed six practice trials (one of each trial type and direction) with feedback. The main block of 438 trials was presented in a single run without breaks to sustain attentional demands.

#### 2.3.3. Rebus Puzzles

Rebus puzzles involved a combination of visual, spatial, verbal, or numerical cues and were used to measure convergent creative problem solving, which was scored as the total number of correct responses (MacGregor and Cunningham 2008). This task included 20 experimental puzzles and 1 practice puzzle. Participants had 30 s to solve each problem (commensurate with prior research; Threadgold et al. 2018), and they pressed the “next” button if they solved a problem before it timed out. Participants entered their answer into a free response text box before moving onto the next problem. All participants were encouraged to try their best to provide an answer to all of the puzzles.

#### 2.3.4. Mindfulness Intervention and Active Control Group

The brief mindfulness intervention led participants through a breath-focused exercise based on classic mindfulness instructions used in Mindfulness-Based Stress Reduction (for a review, see Norris et al. 2018). The session was delivered via a pre-recorded audio track, which participants listened to individually in a quiet, distraction-free room. Instructions guided participants to orient their attention to the present moment with openness and curiosity, encouraging a non-judgemental attitude toward their thoughts and sensations (e.g., “stay open and curious about your experience”). The script followed a continuous structure without breaks, closely modelling the first session of a standard MBSR course (Kabat-Zinn 2017). The content focused specifically on present-moment breath awareness, fostering attentional stability and awareness of internal experiences.

The active control condition consisted of 10 min of audiobook recording of the beginning of “Harry Potter and the Philosopher’s Stone”, narrated by Stephen Fry and delivered via the Audible application. The procedure for the control group ensured participants were sitting quietly in a relaxed state similar to the intervention group, with the only difference being the contents of the audio recording either being mindfulness-focused or not. Thus, we can be confident that any observed differences between the groups could be attributed to the specific characteristics of mindfulness. Participants were instructed to silence their cell phones and any electronic devices prior to the intervention.

#### 2.3.5. Self-Report Scales

The following self-report mindfulness scales were included as control measures to provide an assessment of any potential between-group differences:

(i) The Five Facet Mindfulness Questionnaire (FFMQ; Baer and Oldham 2006) assessed five mindfulness dimensions with 39 items rated from 1 to 5. This scale demonstrates strong internal consistency (Cronbach’s alpha > 0.70) and good construct validity.
(ii) The Multidimensional Assessment of Interoceptive Awareness (MAIA; Mehling et al. 2012) measured present-moment awareness with 37 items rated from 0 to 5. This scale demonstrates reliability coefficients that range from 0.66 to 0.87.
(iii) The Mindful Attention Awareness Scale (MAAS; Brown and Ryan 2003) evaluated attention and awareness with 15 items rated from 1 to 6. This scale has a reliability coefficient of 0.76 and a test–retest reliability of 0.69.

### 2.4. Procedure

All participants were recruited via departmental emailing lists and advertisement posters placed around the University campus. As noted above, participants (*N* = 117) were pseudo-randomly assigned to the mindfulness or control conditions based on their order of registration for the study. Whilst this approach ensured temporal balance in group assignment, we acknowledge that true randomisation (e.g., via a random number generator) would have provided stronger experimental control.

First, intervention participants completed a 10 min mindfulness guidance practice, and control participants listened to an audio book for the same amount of time. Both conditions were in silent laboratory rooms, and participants were alone. Participants then completed each task individually on a laptop, whereby the order of the SART and flanker task was counterbalanced. Participants were provided with instructions for each task and were able to complete practice trials for both the flanker and SART to ensure familiarity with the task and computer. Finally, participants were asked to complete the battery of rebus puzzles on Qualtrics and complete the questionnaires in the order reported above. Ethical approval was obtained from the University of Lancashire Ethics Committee (approval code: PSYSOC463).

## 3. Results

### 3.1. Preliminary Analyses

To ensure that our groups did not differ on baseline dispositional mindfulness, independent *t*-tests were conducted to compare groups in terms of their subjective responses to the mindfulness scales. Groups did not differ at baseline on the FFMQ, *t*(115) = −0.69, *p* = 0.49, or the MAAS, *t*(115) = −0.33, *p* = 0.70. Although the groups did differ significantly at baseline on the MAIA, *t*(115) = −6.24, *p* < 0.001, further analyses of the MAIA subscales revealed that this difference was driven by the self-regulation subscale, on which the intervention group reported significantly higher scores than the control group (*p* = 0.041). The remaining subscales of noticing, attentional regulation, emotional awareness, and body listening or trust were comparable across the groups (all *p*s > 0.05). Given that the self-regulation subscale primarily reflects perceived ability to regulate distress and maintain homeostasis, it is unlikely to influence attentional performance directly or creative problem solving in the tasks used in the present study (i.e., the SART, flanker task, and rebus puzzles). The self-reported mindfulness measures were therefore not included as main variables in the subsequent analyses.

To assess whether prior mindfulness experience moderated task performance, we conducted a series of linear regression analyses examining interaction effects between group (intervention vs. control) and mindfulness experience (experienced vs. naive) across all dependent variables (SART reaction time and accuracy, flanker task accuracy and reaction time in congruent, incongruent, and reversed trials, and rebus puzzle scores). We employed the interactions package to probe interaction effects and generate interaction plots where necessary (version 1.2.0; Long 2024). For all dependent variables, the interaction effects between group and mindfulness experience were not significant (all *p*s > 0.08), indicating that the effects of mindfulness training did not vary based on prior mindfulness experience (see Appendix D).

### 3.2. Data Processing and Analyses

All analyses were conducted using *R* (version 4.4.2). Generalised linear mixed models (GLMMs) were employed to analyse both reaction time and accuracy data across the SART and flanker tasks. GLMMs were chosen to account appropriately for variability associated with both the fixed effects and random interindividual differences among participants (Lo and Andrews 2015). All models were implemented using the *glmer* function from the *lme4* package (version 1.1.36; Bates et al. 2015) with 12,000 iterations (*maxfun*). For accuracy analyses, models used a *binomial* distribution with a *logit* link function. For reaction time analyses, models used a *gamma* distribution with an *identity* link function. Fixed factors were coded using successive differences contrasts, specified with the function *contr.sdif* from the *MASS* package for *R* (version 7.3.61; Venables and Ripley 2002). The significance of fixed effects was assessed using Wald *z*-tests, with *z* > 1.96 considered statistically significant. Details of the random effects structure specification and the trimming procedures applied during model fitting are provided within the *R* scripts included in the data repository.

### 3.3. The SART

For the SART analyses, probe-caught mind wandering was categorised into four mutually exclusive types so that “1” always indicated “on-task thoughts”, “2” always indicated “task-related thoughts”, “3” always indicated “environment-related thoughts”, and “4” always indicated “SITUTs”. Our primary analytical focus was on the overall influence of mind-wandering categories on task performance, captured through the main effects in the GLMMs. We did not include interaction terms involving group or between mind-wandering categories, as our goal was not to examine differential group effects within specific mind-wandering categories, but rather to characterise how each category independently relates to sustained attention performance. This approach reflects a parsimonious modelling strategy that avoids overcomplicating data interpretation with higher-order interactions, which were beyond the scope of this study.

Table 1 presents the frequencies of each mind-wandering category expressed as the proportion of total mind-wandering trials for both the intervention and control groups. Descriptively, the intervention group reported fewer on-task thoughts, but more task-related and environment-related thoughts compared to the control group. SITUTs also appeared less frequent in the intervention group. However, these differences were not formally tested for statistical significance and were instead used to observe changes in sustained attention.

The first GLMM analysed accuracy in the SART (see Table 2). Participants were treated as random effects, while group (intervention vs. control) and mind-wandering types were coded as fixed effects. Frequencies of the four mind-wandering categories, calculated for each participant and collapsed across trials, were included as continuous predictors to assess associations between individual mind-wandering tendencies and accuracy. The intervention group served as the reference category, such that negative coefficients for group indicate higher accuracy in the intervention group relative to the control group. The model showed no significant differences in accuracy between groups (Intervention: *M* = 90.4%, *SD* = 3.1%; Control: *M* = 88.7%, *SD* = 3.4%). Only environment-related thoughts were linked to a significant decrease in accuracy, suggesting attention to external distraction may be particularly disruptive during the SART.

A second GLMM (Model 2) was used to examine reaction times as a function of group and probe-caught mind wandering categories using the same intercept as Model 1. Fixed-effects estimates are presented in Table 3, showing a significant main effect of group. The model indicated that the intervention group responded significantly faster than the control group (Intervention: *M* = 355 ms, *SD* = 45 ms; Control: *M* = 399 ms, *SD* = 48 ms), corresponding to an estimated 44 ms group difference, suggesting that brief mindfulness practice benefits reaction times in the SART.

Regarding mind wandering, higher frequencies of on-task thoughts were significantly associated with faster reaction times. In contrast, higher frequencies of task-related thoughts and SITUTs were significantly associated with slower reaction times, suggesting decreased response efficiency when these types of mind-wandering increased. There was also a marginal trend for environment-related thoughts to be associated with slower reaction times (*p* < 0.10).

### 3.4. The Flanker Task

As with the SART, we examined group differences in flanker performance using two separate GLMMs. Prior to analysis, we excluded trials with incorrect responses due to failure to respond within the 500 ms window. Specifically, 11.5% of errors (1775 trials) were of this latter type. The remaining 88% of errors (15,438 trials) were treated as genuine incorrect responses. We also excluded 5% of trials (2721 trials) with response times under 100 ms, as these likely reflected anticipatory or accidental responses. This left a total of 46,984 trials for analysis.

Descriptive data are presented in Table 4, showing mean accuracy percentages across stimulus types in the flanker task. Accuracy is higher in the intervention group across all stimulus types, with the most notable difference observed in the reversed trials.

Model 3 analysed accuracy in the flanker task. Participants were treated as random intercepts, and group (intervention vs. control) and stimulus type (congruent vs. incongruent vs. reversed) were coded as fixed effects. The intervention group and congruent trials served as the intercepts. The model (see Table 5) revealed a significant main effect of group, with the intervention group demonstrating higher overall accuracy than the control group. There was also a significant main effect of stimulus type, as accuracy was lower on incongruent compared to congruent trials, while reversed trials did not differ significantly from incongruent trials. The model also revealed significant interaction terms between group and stimulus type, indicating that the effect of group varied dependent upon stimulus type, warranting further investigation.

To investigate the significant interaction between group and stimulus type on accuracy, we conducted pairwise comparisons using estimated marginal means derived from the GLMM. A new variable name was created to combine group and stimulus type, taking the design of the study from a 2 (group) × 3 (stimulus type) design to a 1 × 6 design (intervention-congruent, intervention-incongruent, intervention-reversed, control-congruent, control-incongruent, and control-reversed). As shown in Table 6, accuracy on congruent trials was significantly higher in the mindfulness group compared to the control group. Descriptive data in Table 4 confirm this finding, suggesting better performance on non-conflict trials in the intervention group relative to the control group. On reversed trials, the intervention group also showed significantly higher accuracy than the control group, indicative of improved conflict monitoring and attentional inhibition following brief mindfulness practice. However, no significant difference in accuracy was observed between groups on incongruent trials (*p* > 0.05).

The magnitude of the between-group differences for congruent trials (difference of 3% accuracy) and reversed trials (difference of 9% accuracy) suggests that mindfulness was more beneficial for trials requiring conflict monitoring in comparison to congruent trials that do not require conflict-monitoring, but which presumably benefit from a degree of sustained attention. However, incongruent trials also require aspects of conflict monitoring, making these findings difficult to explain. Possible explanations for this apparent inconsistency are proposed in Section 4.2.

The fourth GLMM (Model 4) examined reaction times as a function of group and stimulus type, using the same intercepts as Model 3. Mean reaction times across stimulus type for both groups are shown in Table 7. As expected, the mean reaction time collapsed across stimulus type was faster in the intervention group relative to the control group (Intervention: *M* = 474 ms, *SD* = 16 ms; Control: *M* = 480 ms, *SD* = 25 ms), suggesting individuals who engaged with the mindfulness intervention were able to respond more quickly to all flanker stimuli. The descriptive data indicate that the intervention group were faster at responding on congruent, incongruent and reversed trials. The largest difference in reaction times was seen for congruent trials, where the mindfulness group were 9 ms faster on average compared to the control group.

GLMM results for Model 4 are shown in Table 8. The significant main effect of group showed that, overall, the intervention group responded significantly faster than the control group across all trial types. The significant main effect of stimulus type indicated that reaction times varied significantly across trial types. Reaction times were significantly slower for incongruent trials compared to congruent ones (*p* < 0.001), and for reversed trials compared to congruent ones (*p* < 0.001). The difference between incongruent and reversed trials was also significant (*p* = 0.042), where reaction times on reversed trials were significantly slower than on incongruent trials, confirming the expected hierarchy of trial difficulty. The model also revealed a significant interaction between group and stimulus type, indicating that the effect of group on reaction times varied according to stimulus type, warranting follow-up analyses.

To examine further the interaction between group and stimulus type, pairwise comparisons were conducted using the new combined factor described above. Reaction times were compared between groups for each stimulus type, and the results are presented in Table 9. There was a significant difference between groups for reaction times on all trial types. The mindfulness group were significantly faster at responding to congruent trials (difference of 9 ms), incongruent trials (difference of 5 ms), and reversed trials (difference of 3 ms) relative to the control group.

### 3.5. Rebus Puzzles

Response accuracies for rebus puzzles were high across groups (Intervention, *M* = 8.53, *SD* = 4.74; Control, *M* = 8.01, *SD* = 4.22). A multiple linear regression analysis was conducted to examine predictors of rebus puzzle accuracy. The predictors included in the regression model were as follows: (i) group (intervention vs. control); (ii) SART reaction times; (iii) accuracy and reaction times across different flanker stimulus types (congruent, incongruent, reversed); and (iv) mindfulness questionnaire scores (FFMQ, MAAS, MAIA). The model included all predictors simultaneously. Results, as presented in Table 10, indicate that the overall model was not statistically significant, *F*(11, 96) = 0.78, *p* = 0.663, and accounted for only 8.2% of the variance in rebus accuracy (adjusted *R*^2^ = −0.024). None of the predictors reached conventional significance levels of 0.05 (all *p*s *>* 0.10*)*. These results suggest that, within the current sample, rebus performance was not strongly predicted by the mindfulness intervention. In addition, rebus performance was also not predicted by reaction time on the SART, or reaction time or accuracy on the flanker task. Furthermore, rebus performance was also not predicted by any of the self-reported mindfulness scores.

Mediation analyses were undertaken to assess in more detail the potential mechanisms that may mediate the relationship between mindfulness and convergent thinking. The first path assessed the relationship between group (intervention vs. control), accuracy in the flanker task, and convergent thinking scores (defined by accuracy on rebus puzzles). Accuracy data were collapsed across flanker stimulus types because performance on specific stimulus types did not significantly predict rebus accuracy in the multiple regression described above, hence, there was no justification to examine the mediating effects of a specific stimulus type. Data were screened for multivariate outliers, leverage and influence prior to the analysis; no cases were removed.

**Figure 4:**
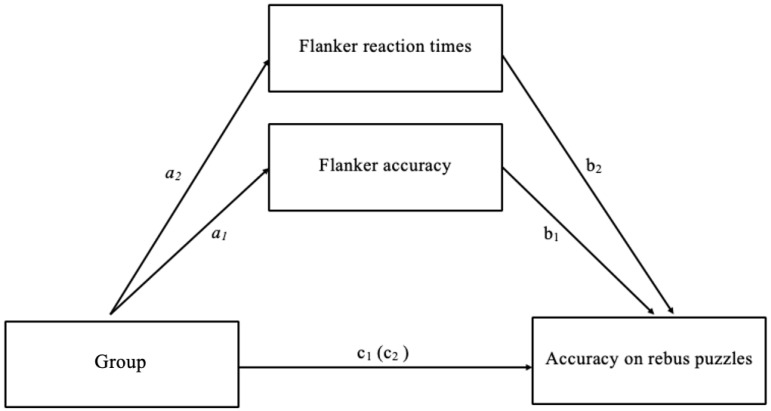
Diagram of the mediated relationships between group, rebus puzzle accuracy, and both flanker accuracy and flanker reaction time measures. *Note*. Pathway 1 (a1, b1) assesses flanker accuracy as a mediator between group and rebus accuracy. Pathway 2 (a2, b2) examines flanker reaction time as the mediator between group and rebus accuracy.

First, using steps described by Baron and Kenny (1986), group was identified as a significant predictor of accuracy in the flanker task (the *a*_1_ pathway). The intervention group showed higher accuracy than the control condition, *t*(114) = −2.45, *p* = 0.02. Second, group was used to predict the scores in rebus puzzles, which showed no significant effect of group on convergent thinking *t*(114), = −1.35, *p* = 0.18 (the c_1_ pathway). Third, the relationship between the accuracy in the flanker task and rebus puzzles was examined controlling for group. Here, the rebus puzzle scores were not significantly related to accuracy scores in the flanker task *t*(114) = −1.41, *p* = 0.16 (the b_1_ pathway). Lastly, the mediated relationship between group condition and rebus puzzle scores was examined for a drop in prediction when the mediator was added into the model. Mediation was not found, showing that the relationship between the group and rebus accuracy was not significant after controlling for accuracy in flanker scores *t*(114) = −1.35, *p* = 0.79. The Sobel test was used to determine that the effect was not significantly greater than 0, *Z* = 1.18, *p* = 0.76 (see Pathway 1 in Figure 4).

A second mediation analysis was undertaken using flanker reaction times as the mediator, again collapsed across stimulus types. The group condition showed faster reaction times in the intervention group compared to the control group, *t*(114) = −0.72, *p* < 0.001 (the *a*_2_ pathway). Group did not significantly predict performance on the rebus puzzles, *t*(114), = −1.35, *p* = 0.79 (the c_2_ pathway). Third, the relationship between the reaction times in the flanker task and rebus puzzles was examined while controlling for group. Here, the rebus puzzle scores were not significantly related to reaction times in the flanker task *t*(114) = 5.64, *p* = 0.26 (the b_2_ pathway). Lastly, the mediated relationship between group and rebus accuracy was examined for a drop in prediction when the mediator was added into the model. Mediation was not found, showing that the relationship between group and rebus accuracy was not significant after controlling for reaction times in flanker scores *t*(114) = −1.42, *p* = 0.69 (see Pathway 2 in Figure 4).

## 4. Discussion

Mindfulness practice is generally associated with enhanced cognition (e.g., Chiesa et al. 2011; Quaglia et al. 2019) with specific advantageous outcomes for sustained attention and attentional inhibition (e.g., Jha et al. 2007; Tang et al. 2015). Convergent creative problem solving relies heavily on these types of attentional processes to both generate and evaluate ideas (Zhang et al. 2020). Despite this theoretical overlap, there remains a lack of clarity regarding the link between mindfulness—particularly short-term practice—and convergent thinking. The current study examined the impact of brief mindfulness practice on sustained attention using the SART, on attentional inhibition using the flanker task, and on creative problem-solving using rebus puzzles.

### 4.1. Main Results

Across two attention tasks, results indicated that a brief mindfulness intervention (10 min) enhanced sustained attention and attentional inhibition. Participants in the mindfulness group showed significantly faster response times on the SART without compromising accuracy and reported fewer task-unrelated thoughts. On the flanker task, the mindfulness group also had higher accuracy across all trial types, and quicker reaction times across congruent and reversed trials. However, contrary to our predictions, there was no significant difference in convergent thinking performance with rebus puzzle between the mindfulness and control groups. Furthermore, mediation analyses showed no evidence that the attention measures explained variability in performance on the rebus puzzles. These findings suggest that while sustained attention and attentional inhibition can improve with brief mindfulness training, such benefits do not translate into enhanced convergent thinking in the form of rebus puzzle solving.

### 4.2. Theoretical and Practical Implications

#### 4.2.1. Mindfulness and Sustained Attention

As expected, brief mindfulness practice was beneficial for sustained attention, such that on the SART, the mindfulness group demonstrated faster reaction times alongside fewer task-irrelevant, mind-wandering thoughts. Accuracy rates were comparable across both groups, potentially due to the simple nature of the task leading to ceiling effects, which are often observed when using the SART in healthy, neurotypical populations (e.g., Schepers et al. 2023). Usually, though, accuracy ceiling rates are often accompanied by increased reaction times, indicating an accuracy–reaction time trade-off (Wilson et al. 2016). The fact that the mindfulness group demonstrated significantly faster reaction times while maintaining very high accuracy rates demonstrates the magnitude of the effect that brief mindfulness practice can have on sustained attention, which is likely due to less interference of task-irrelevant, distracting thoughts, as evidenced by a clear reduction in SITUTs in the mindfulness group.

Contrary to prior research (e.g., Mrazek et al. 2013; Schooler et al. 2014), participants in the mindfulness group reported more mind-wandering thoughts overall compared to the control group. However, a closer examination of the types of mind-wandering revealed that the mindfulness group experienced more environment-related and task-related thoughts, indicating heightened present-moment awareness, aligning with the core aims of mindfulness practice (Kabat-Zinn 1982). Importantly, these increases in environment-related and task-related thoughts appeared not to impact reaction times negatively on the SART, as evidenced by significantly faster reaction times in the mindfulness group overall. Given that SITUTs were the only type of mind-wandering thoughts to reduce in response to mindfulness practice, this may point to the fact that when SITUTs are present, they may be particularly detrimental to reaction times on tasks requiring sustained attention.

#### 4.2.2. Mindfulness and Attentional Inhibition

As predicted, participants who completed a brief mindfulness practice demonstrated better performance on the flanker task compared to those in the control group. Specifically, the mindfulness group showed significantly faster reaction times across all trial types, as well as higher accuracy on both congruent and reversed trials. These findings support the effectiveness of a short mindfulness intervention (i.e., a single 10 min session) in enhancing attentional inhibition and promoting more efficient use of attentional resources, aligning with prior research (e.g., Norris et al. 2018).

Typically, congruent trials in the flanker task serve as a baseline condition and are not expected to yield differences between groups, as they involve minimal cognitive conflict (e.g., Jha et al. 2007; Norris et al. 2018). However, in the present study, participants in the mindfulness group responded both faster and more accurately on these trials compared to controls. This improvement in a low-conflict condition may reflect an overall enhancement in sustained attention, allowing for quicker and more precise responses even when task demands are low, further supporting our conclusions from the SART. For conflict trials (i.e., incongruent and reversed), the mindfulness group also responded faster than the control group, suggesting improved ability to inhibit interference from distracting flankers. Interestingly, accuracy improvements were observed only on reversed trials, not incongruent trials. Whilst participants in the mindfulness group responded more quickly on both types of conflict trials, only reversed trials showed a significant accuracy advantage. This pattern suggests that mindfulness may be especially beneficial in conditions requiring not just attentional inhibition, but also greater cognitive flexibility, such as overriding a well-learned response pattern. We outline this cognitive flexibility account below, alongside another possible interpretation of the data based on the concept of information salience.

In terms of the cognitive flexibility account of the accuracy advantage for reversed trials over incongruent trials following mindfulness practice, a possible interpretation is that reversed trials place greater demands on cognitive flexibility due to their task-switching nature, as they require disengaging from a habitual response and applying a new rule (i.e., reversing the usual response mapping; Chen et al. 2022). Mindfulness is associated with reduced perseveration and increased flexibility (McBride and Greeson 2023), which could explain the superior performance of the mindfulness group on reversed trials, in particular. In contrast, congruent trials are straightforward and require minimal flexibility, while incongruent trials, although more challenging, still operate within a consistent response framework that can be managed either automatically or through practiced inhibitory control. The need to override automatic responses in reversed trials likely engages deliberate control processes, an area where mindfulness may exert stronger effects (Chiesa et al. 2011).

Alternatively, in line with a salience-based theory, visually distinct stimuli (e.g., brightly coloured or novel features) can capture attention regardless of task goals (Anderson et al. 2011; Geyer et al. 2008). In our flanker task, reversed trials were presented in red, contrasting with the green used in congruent and incongruent trials, making them more perceptually salient. These trials also required an opposite button press, adding an additional layer of conflict. The combination of visual distinctiveness and increased cognitive demand likely drew more attention, as well as mindfulness practice, by fostering heightened awareness of task-relevant stimuli (Quaglia et al. 2019), may have amplified participants’ ability to respond efficiently and accurately to these salient, high-conflict trials. In contrast, congruent trials involve no conflict, and while incongruent trials require attentional inhibition, they lack the perceptual novelty of reversed trials. This may explain why the benefits of mindfulness were most pronounced in reversed trials: the enhanced present-moment awareness cultivated by mindfulness may be most useful when processing both salient and cognitively demanding stimuli. These two aforementioned accounts offer plausible explanations of our data that future research may arbitrate between.

#### 4.2.3. Mindfulness and Convergent Thinking

Despite extensive research supporting a positive relationship between mindfulness and creativity (e.g., Baas et al. 2014; Ostafin and Kassman 2012), the current study found no significant difference in rebus puzzle accuracy between the mindfulness group and the control group. Our hypothesis, that brief mindfulness practice would enhance convergent thinking, was therefore not supported. Additionally, there was no evidence of a mediating effect of improved attention on rebus accuracy scores. These findings suggest that, at least in the context of rebus puzzles, convergent thinking may not profit from the enhanced sustained attention or attentional inhibition afforded by brief mindfulness interventions.

The type of creativity assessed is an important factor when interpreting these results. Previous meta-analyses (e.g., Hughes et al. 2023; Lebuda et al. 2016) have highlighted that mindfulness tends to benefit tasks involving convergent rather than divergent thinking. To reiterate, rebus puzzles are a hybrid form of convergent problem solving that can be approached either analytically or through insight-based “Aha!” moments (Jung-Beeman et al. 2004; Salvi et al. 2021). Solving rebus puzzles has been thought to involve multiple levels of restructuring to reach a solution (MacGregor and Cunningham 2008). Little research has investigated the effect of short mindfulness practice on this specific task type. The current findings support the notion that brief mindfulness practice does not enhance convergent thinking in a task that requires complex restructuring. We outline three possible explanations for this outcome:

First, the difficulty of the rebus puzzles was not systematically controlled or manipulated in the current study. Given that the attentional benefits of mindfulness are often more apparent in tasks with higher cognitive demands (Norris et al. 2018), it is possible that the puzzles that we used did not meet the requisite threshold whereby attentional enhancements through mindfulness practice could facilitate solution generation. This possibility is supported by the fact that rebus accuracy was found to be relatively high (greater than 80%) across groups in our study. Thus, although brief mindfulness practice successfully enhanced sustained attention and attentional inhibition in the SART and flanker task, respectively, such attentional enhancements did not translate to measurable gains in convergent thinking, possibly due to the low demand of the rebus puzzles used in the study.

Second, the duration of the mindfulness intervention may have been insufficient to impact convergent thinking. Although the 10 min session was effective in improving attentional processes, convergent thinking may require more sustained interventions to yield measurable changes. Previous research suggests that mindfulness can improve creative performance by enhancing executive control or increasing efficiency in relevant brain regions (e.g., the Default Mode Network—see Garrison et al. 2015; or the Executive Control Network—see Taren et al. 2017). However, the short intervention used here may not have produced such changes at a level sufficient to influence convergent thinking.

Third, the cognitive processes underlying rebus puzzles may differ from those engaged in other forms of convergent thinking. Traditional models propose that creativity involves both generation and evaluation stages, with executive control playing a key role in the latter (Kenett et al. 2018). However, other research (Richardson et al. 2018) suggests that rebus puzzles may not involve an evaluative phase. Instead, when a solution emerges, it is immediately obvious whether it is correct or not, rendering the attentionally demanding evaluation stage unnecessary. If executive control is *not* engaged in evaluating the solutions to rebus puzzles, then improvements in attentional inhibition and cognitive flexibility in response to brief mindfulness practice may not influence performance, aligning with the null findings observed in the current study.

### 4.3. Limitations and Recommendations for Future Research

One of the main challenges in mindfulness research is the heterogeneity of mindfulness practices and interventions, which makes it difficult to draw generalizable conclusions—particularly when comparing short-term interventions with long-term training or experienced meditators (Van Dam et al. 2018). Although the present study employed a brief mindfulness session modelled on standardised definitions such as Kabat-Zinn’s (1982), this short-term format may not fully capture the depth or sustained effects of mindfulness practice over time. As a result, caution is warranted when extrapolating findings to broader mindfulness applications. To address common criticisms regarding expectancy and placebo effects (e.g., Bishop 2002; Baer 2003), the current study included an active control group, which was matched in duration, but differed in its content. This design aimed to isolate the specific cognitive effects of mindfulness while minimising confounds related to participant engagement or demand characteristics. Although this approach improves methodological rigour, further refinements to control conditions may enhance future research. For instance, matching control tasks more closely in structure but not in mindfulness-relevant processes, such as attention to breath or non-judgemental awareness, could provide a more stringent test of mindfulness-specific benefits (Norris et al. 2018). One possible avenue is the inclusion of breathing or relaxation exercises in control groups, which can help account for the calming effects of focused attention without introducing mindfulness-specific attitudes (Noone and Hogan 2018). However, identifying the most appropriate and theoretically neutral control task remains a significant methodological challenge.

Additionally, participants were pseudo-randomly assigned to the mindfulness and control conditions based on the order of their registration for the study. Although this approach ensured temporal balance in group assignment, we acknowledge that true randomisation would have provided stronger experimental control and reduced the risk of allocation bias. Furthermore, no baseline assessments of convergent thinking or attention were conducted prior to the intervention, limiting our ability to confirm cognitive equivalence between groups. This restricts causal interpretations of the effects observed, and future research would benefit from incorporating pre-test post-test designs, alongside an active control group, to strengthen inferences regarding mindfulness-related cognitive changes. We additionally note that the majority of our sample (69%) identified as White British. Owing to the limited ethnic diversity in our participant pool, we were unable to assess potential racial or cultural moderators of mindfulness outcomes, which limits the generalisability of our findings and highlights the need for future studies to examine mindfulness effects in more diverse and representative samples.

Finally, although our study focused primarily on the effects of mindfulness on convergent thinking, consistent with meta-analytic findings (Hughes et al. 2023) demonstrating that mindfulness interventions tend to yield stronger benefits for convergent rather than divergent thinking, it is important to acknowledge the potential for mindfulness to also enhance divergent thinking. Previous research suggests that mindfulness may facilitate flexible and novel idea generation, which is central to divergent thinking (Giancola et al. 2024). Future research would do well to extend the current findings by incorporating a broader range of creativity assessments, including tasks such as the Alternative Uses Task (Guilford 1967) and Urban’s figural test (Jellen and Urban 1986), to more comprehensively investigate how mindfulness impacts divergent thinking.

## 5. Conclusions

The current empirical study has provided evidence that brief mindfulness practice can enhance attentional control processes. Specifically, participants in the mindfulness group exhibited faster reaction times on the SART, alongside fewer task-irrelevant mind-wandering thoughts, reflecting improved sustained attention. In addition, we found faster reaction times and greater accuracy on the flanker task in response to brief mindfulness practice, with a particular benefit to conflict trials, indicative of enhanced attentional inhibition. Consistent with previous research (e.g., Lin et al. 2019; Norris et al. 2018; Wimmer et al. 2020), our results suggest that short-term mindfulness interventions can yield attention-related benefits, particularly in tasks requiring sustained attention and attentional inhibition.

Unexpectedly, these latter attentional improvements did not translate into enhanced performance on a convergent thinking task. No group differences were observed in rebus puzzle accuracy, nor did sustained attention or attentional inhibition mediate rebus puzzle performance. These findings challenge the assumption that improvements in sustained attention and attentional inhibition directly support all forms of creative cognition. We propose that some insight problems, such as rebus puzzles, may bypass executive evaluation, thus limiting the potential impact of improved attention afforded by mindfulness. Therefore, although our findings support the benefits of brief mindfulness for attentional control, they also suggest that such benefits may not necessarily generalise to convergent thinking tasks.

## Figures and Tables

**Table 1:** The frequency of each mind wandering category (expressed as a percentage of total mind wandering trials) during the SART.

Mind Wandering Category	Intervention (*SD*)	Control (*SD*)
On-task thoughts	0.52 (0.31)	0.58 (0.31)
Task-related thoughts	0.22 (0.35)	0.17 (0.37)
Environment-related thoughts	0.18 (0.31)	0.12 (0.26)
SITUTs	0.08 (0.32)	0.13 (0.29)

**Table 2:** Model 1: Fixed-effects estimates from the GLMM on accuracy on the SART.

Accuracy					
Factor	*β*	*SE*	*z*-Value	Sig	95% CI
Intercept	3.21	0.424	7.60	***	[2.38, 4.04]
Group	−0.42	0.584	−0.721		[−1.57, 0.73]
On-task thoughts	0.78	0.442	1.76		[−0.09, 1.65]
SITUTs	−0.93	0.518	−1.80		[−1.95, 0.09]
Task-related thoughts	−0.71	0.661	−1.07		[−2.01, 0.59]
Environment-related thoughts	−0.98	0.410	−2.37	*	[−1.79, −0.17]

*Note.* SITUTs is an abbreviation for stimulus independent task unrelated thoughts. * *p* < 0.05, *** *p* < 0.001. Empty cells in the significance column imply no significant result.

**Table 3:** Model 2: Fixed-effects estimates from the GLMM on reaction times on the SART.

Reaction Time					
Factor	β (ms)	*SE*	*t*-Value	Sig	95% CI
Intercept	281.26	4.97	56.59	***	[271.51, 291.01]
Group	−44.25	3.38	−13.09	***	[−50.87, −37.63]
On-task thoughts	−40.37	2.02	−2.01	***	[−44.32, −36.42]
SITUTs	12.25	6.04	2.03	*	[0.39, 24.11]
Task-related thoughts	30.13	6.01	5.01	***	[18.33, 41.93]
Environment-related thoughts	26.81	14.51	1.85	**.**	[−1.63, 55.25]

*Note.* SITUTs is an abbreviation for stimulus independent task unrelated thoughts. * *p* < 0.05, *** *p* < 0.001, **.** *p* < 0.1.

**Table 4:** Mean accuracy percentages across stimulus types in the flanker task.

Stimulus Type	Intervention (*SD*)	Control (*SD*)
Collapsed across trials	71 (0.46)	66 (0.47)
Congruent trials	83 (0.40)	80 (0.40)
Incongruent trials	62 (0.48)	61 (0.49)
Reversed trials	67 (0.47)	58 (0.48)

**Table 5:** Model 3: Fixed-effects estimates from the GLMM on accuracy on the flanker task.

Accuracy					
Factor	*β*	*SE*	*z*-Value	Sig	95% CI
(Intercept)	0.934	0.096	9.71	***	[0.75, 1.12]
Group (intervention vs. control)	−0.64	0.19	−3.39	***	[−1.01, −0.27]
Stimulus type (incongruent–congruent)	−1.042	0.102	−10.228	***	[−1.24, −0.84]
Stimulus type (reversed–incongruent)	−0.17	0.26	−0.64		[−0.69, 0.35]
Group (mindfulness) × Stimulus type (incongruent-congruent)	−0.516	0.201	−2.56	*	[−0.91, −0.12]
Group (mindfulness) × Stimulus (reversed–incongruent)	−0.83	0.37	−2.24	*	[−1.56, −0.10]

*Note.* * *p* < 0.05, *** *p* < 0.001.

**Table 6:** Between-group effects on accuracy on the flanker task.

Between-Group Effects				
Factor	*β*	*z*-Value	Sig	Cohen’s *d*
Intervention congruent—Control congruent	1.053	6.53	**	0.60
Intervention incongruent—Control incongruent	0.231	1.163		0.11
Intervention reversed—Control reversed	1.68	9.67	***	0.89

*Note.* ** *p* < 0.01, *** *p* < 0.001. Empty cells in the significance column imply no significant result.

**Table 7:** Mean reaction times across stimulus types in the flanker task.

Stimulus Type	Intervention (*SD*)	Control (*SD*)
Collapsed across stimulus type	474 (16)	480 (25)
Congruent	439 (16)	448 (16)
Incongruent	488 (19)	493 (22)
Reversed	495 (19)	498 (22)

**Table 8:** Model 4: Fixed-effects estimates from the GLMM on reaction times on the flanker task.

Reaction Time					
Factor	*β*	*SE*	*t*-Value	Sig	95% CI
(Intercept)	452.78	0.657	6.74	***	[451.49, 454.07]
Group (intervention vs. control)	9.41	0.659	14.27	***	[8.12, 10.70]
Stimulus type (incongruent–congruent)	29.98	0.735	40.80	***	[28.54, 31.42]
Stimulus type (reversed–incongruent)	6.24	0.775	8.05	*	[4.72, 7.76]
Group x Stimulus type (incongruent–congruent)	26.44	0.872	3.32	***	[24.73, 28.15]
Group x Stimulus type (reversed–incongruent)	4.18	0.91	4.62	***	[2.40, 5.96]

*Note.* * *p* < 0.05, *** *p* < 0.001, empty cells in the significance column imply no significant result.

**Table 9:** Between-group effects on reaction times on the flanker task.

Between-Group Effects				
Factor	*β*	*z*-Value	Sig	Cohen’s *d*
Control congruent–intervention congruent	9.22	2.55	**	0.24
Control incongruent–intervention incongruent	5.03	7.43	***	0.69
Control reversed–intervention reversed	3.14	4.97	**	0.40

*Note.* *** *p* < 0.001, ** *p* < 0.01.

**Table 10:** Multiple linear regression predicting rebus accuracy based on group (intervention vs. control), flanker performance (accuracy and reaction times) across stimulus types, SART reaction times, and self-report mindfulness scores.

Predictor	Estimate (β)	Std. Error	*t*	*p*
(Intercept)	14.08	7.46	1.89	.062
Group (Control)	−0.26	1.05	−0.25	.804
Accuracy (Congruent)	3.42	3.73	0.92	.361
Accuracy (Incongruent)	−4.31	2.25	−1.92	.058
Accuracy (Reversed)	2.54	3.01	0.85	.400
RT (Congruent)	−0.008	0.013	−0.59	.554
RT (Incongruent)	0.016	0.010	1.60	.113
RT (Reversed)	−0.010	0.009	−1.11	.270
FFMQ	−1.46	1.67	−0.87	.386
MAAS	−0.10	0.64	−0.15	.878
MAIA	−1.45	2.32	−0.62	.534
SART_RT	0.007	0.007	1.08	.282

*Note.* Model R^2^ = 0.082, adjusted R^2^ = −0.024, F(11, 96) = 0.78, *p* = 0.663.

## Data Availability

All data and research materials have been made publicly available at The Open Science Framework (https://osf.io/cgytx/?view_only=919eea87b39045ac95ff0943fd6a4253, accessed on 2 August 2025). This project was not preregistered.

## Appendix A. Demographic Composition of the Mindfulness and Control Groups

**Table A1:** Summary of the demographic composition of the mindfulness and control groups.

Variable	Mindfulness Group (*n* = 60)	Control Group (*n* = 57)
Female	46	43
Male	12	14
Undisclosed gender	2	0
Mean age (*SD*)	23.02 (7.07)	22.09 (5.20)
Ethnicity: White British	38	43
Right-handed	54	50
Left-handed	6	7
Prior mindfulness experience	15	12

## Appendix B. Demographic Comparisons Between the Mindfulness and Control Groups

**Table A2:** Demographic comparison tests between the mindfulness and control groups.

Demographic Variable	Test Type	Test Statistic	*p*-Value
Age	*t*-test	*t*(115) = 0.81	0.42
Gender (male/female)	Chi-square	χ^2^(2) = 2.18	0.336
Mindfulness experience (yes/no)	Chi-square	χ^2^(1) = 0.26	0.612
Handedness (right/left)	Chi-square	χ^2^(1) = 0.15	0.695

## Appendix C. Assessing Congruency Effects in Reversed Trials

We did not distinguish between congruency in reversed trials because, under the reversed response mapping, trials labelled as “incongruent” would still feature four out of five arrows pointing in the same direction, making them perceptually similar to congruent trials and thus unlikely to differ meaningfully in processing. To check this, we compared performance between reversed incongruent (RI) and reversed congruent (RC) trials.

A logistic regression was conducted to examine the effect of stimulus type (RI vs. RC) on accuracy (binary outcome). The model revealed no significant effect of stimulus type, indicating that accuracy did not differ between the two conditions, *b* = 0.05, *SE* = 0.03, *z* = 1.54, *p* = 0.13. The odds ratio for accuracy in the RC condition compared to RI was 1.05 (95% CI [0.99, 1.12]), suggesting no meaningful change in the likelihood of a correct response. An analysis of variance (ANOVA) was conducted to test for differences in reaction times between the stimulus type conditions. Because of violations of normality assumptions (Shapiro–Wilk test *p* < 0.001), a nonparametric Wilcoxon rank-sum test was performed instead. There was no significant difference in reaction times between the RI and RC trials (*p* = 0.38). These results support our decision to collapse across congruency in reversed trials, as performance did not differ meaningfully between reversed incongruent and reversed congruent conditions.

## Appendix D. Assessing the Impact of Prior Mindfulness Experience on Performance

A series of linear regression analyses were conducted to examine the effects of group (intervention vs. control) and prior experience (experienced vs. naive) on performance across multiple cognitive tasks. The analyses assessed whether there were significant interaction effects between group and experience on the following dependent variables: (i) SART reaction time; (ii) rebus accuracy; (iii) flanker task accuracy for all stimulus types (congruent, incongruent, reversed); and (iv) flanker task reaction times for all stimulus types (congruent, incongruent, reversed). All analyses were performed using *R* (version 4.3.2), with the interactions package employed to examine interaction effects. The interact_plot() function was initially used but was found to be less suitable for models with categorical predictors. Therefore, the cat_plot() function from the interactions package was used to visualise appropriately the categorical interactions. The interaction effects for each dependent variable were examined. Full regression outputs are presented in Table 1.

**Table A3:** Summary of regression results for the group and mindfulness experience interaction.

Dependent Variable	Estimate	Std. Error	*t*-Value	*p*-Value
SART reaction time	16.35	31.98	0.51	0.61
Rebus accuracy	3.66	2.05	1.79	0.08
Flanker accuracy (congruent)	0.09	0.07	1.31	0.19
Flanker accuracy (incongruent)	−0.02	0.13	−0.14	0.89
Flanker accuracy (reversed)	0.03	0.11	0.28	0.78
Flanker reaction time (congruent)	21.25	31.91	0.67	0.51
Flanker reaction time (incongruent)	28.11	44.64	0.63	0.53
Flanker reaction time (reversed)	14.60	46.96	0.31	0.76

The primary focus was to detect potential interaction effects between group and mindfulness experience on task performance. Across the tasks, the interaction effects were not statistically significant (all *p*s > 0.08), indicating that the influence of mindfulness practice (versus the control condition) did not significantly differ based on participants’ prior mindfulness experience for any of our variables. Therefore, prior experience was not included as a factor in the main analyses.
